# High Fat Diet-Induced miR-122 Regulates Lipid Metabolism and Fat Deposition in Genetically Improved Farmed Tilapia (GIFT, *Oreochromis niloticus*) Liver

**DOI:** 10.3389/fphys.2018.01422

**Published:** 2018-10-05

**Authors:** Jun Qiang, Yi Fan Tao, Jing Wen Bao, De Ju Chen, Hong Xia Li, Jie He, Pao Xu

**Affiliations:** ^1^Key Laboratory of Freshwater Fisheries and Germplasm Resources Utilization, Ministry of Agriculture, Freshwater Fisheries Research Center, Chinese Academy of Fishery Sciences, Wuxi, China; ^2^Wuxi Fisheries College, Nanjing Agricultural University, Wuxi, China

**Keywords:** miR-122, genetically improved farmed tilapia, stearoyl-CoA desaturase, high fat diet, lipid metabolism

## Abstract

The liver is an important organ for the regulation of lipid metabolism. In genetically improved farmed tilapia (GIFT, *Oreochromis niloticus*), fat deposition in the liver occurs when they are fed high-lipid diets over a long term. This can affect their growth, meat quality, and disease resistance. MicroRNAs (miRNAs) are known to be crucial regulatory factors involved in lipid metabolism; however, the mechanism by which they regulate lipid deposition in GIFT remains unclear. Comparative miRNA expression profiling between GIFT fed a normal diet and those fed a high-lipid diet showed that miR-122 is closely related to lipid deposition. Using miR-122 as a candidate, we searched for a binding site for miR-122 in the 3′-untranslated region (UTR) of the stearoyl-CoA desaturase gene (*SCD*) using bioinformatics tools, and then confirmed its functionality using the luciferase reporter gene system. Then, the regulatory relationship between this miRNA and its target gene *SCD* was analyzed using real-time polymerase chain reaction (qRT-PCR) and western blotting analyses. Last, we investigated the effect of the loss of miR-122 expression on lipid metabolism in GIFT. The results showed that a sequence in the 3′-UTR region of *SCD* of GIFT was complementary to the miR-122 seed region, and there was a negative relationship between the expression of miRNA and *SCD* expression. Inhibition of miR-122 up-regulated *SCD*, increased the expression of fat synthesis-related genes, increased hepatic triglyceride and cholesterol contents, and promoted weight gain in fish. Our results showed that miR-122 targets *SCD* to mediate hepatic fat metabolism. These results provide new insights for the prevention and treatment of fatty liver disease in GIFT.

## Introduction

Liver tissues are enriched in microRNA-122 (miR-122). This miRNA accounts for 70 and 52% of the total miRNAs in the liver of adult mice and humans, respectively (Bandiera et al., 2015). miR-122 plays important roles in promoting liver development and differentiation and in maintaining its function and balance. The expression of miR-122 in mouse embryonic liver and human hepatoma cell lines is associated with liver-enriched transcription factors (Xu et al., 2010; Laudadio et al., 2012; Deng et al., 2014). Especially during liver development, the mutual restriction between miR-122 and liver-enriched transcription factors helps to maintain the balance between cell proliferation and differentiation in hepatocytes and biliary epithelial cells (Xu et al., 2010; Laudadio et al., 2012). miR-122 also plays an important role in regulating liver cholesterol and fatty acid metabolism. Gene chip expression analyses have shown that the inhibition of miR-122 results in decreased expression of many genes encoding enzymes related to cholesterol synthesis, including 3-hydroxy-3-methyl-glutaryl-CoA reductase, which is the rate-limiting enzyme in cholesterol synthesis. miR-122 may affect cholesterol synthesis by silencing the activity of the transcriptional repressor of 3-hydroxy-3-methyl-glutaryl-CoA reductase (Krützfeldt et al., 2005; Esau et al., 2006). In addition, miR-122-deficient mice displayed reduced plasma triglyceride and cholesterol levels and fat deposition (Esau et al., 2006).

In recent years, tilapia aquaculture has become more intensive and non-parasitic fish diseases caused by environmental conditions and other factors have become increasingly serious. Such diseases can result from dietary imbalances, such as long-term feeding of high-protein, high-fat, or high-carbohydrate diets. These diets can result in damage to the liver, kidney, heart, and other metabolically regulated tissues, leading to fat deposition in liver cells and various nutritional diseases, among which fatty liver is the most serious (Qiang et al., 2017a). However, few studies have focused on the molecular biology of fat deposition. In this study, we selected genetically improved farmed tilapia (GIFT, *Oreochromis niloticus*), which had been bred using family breeding methods and assessed using the best linear unbiased prediction breeding evaluation method. At present, aquacultured GIFT accounts for 75% of the total aquacultured tilapia produced in China. Therefore, research on GIFT is important from both biological and economic perspectives.

In our previous study, we used expression profiling to analyze differentially expressed miRNAs between GIFT fed a high-fat diet and those fed a normal diet. The expression levels of miR-122, miR-29a, miR-205-5p, and miR-34a differed significantly between the high-fat diet group and the normal-fat diet group (Tao et al., 2017). A cluster analysis of miR-122 showed that its regulatory pathways were mainly enriched in the metabolism of sugars, lipids, and proteins in the liver, suggesting that miR-122 may participate in the regulation of some fat metabolism processes in GIFT liver. Based on the whole genome sequence of tilapia^1^, we used bioinformatics software (TargetScan5.2^2^ and the miRanda v3.3a toolbox^3^) to detect the potential target genes of the differentially expressed miRNAs, using the default parameters and cut-offs (score *S* ≥ 140; energy *E* ≤-7.0 kcal mol^-1^) (Betel et al., 2010). As a result of those analyses, we predicted that the stearoyl-CoA desaturase gene (*SCD*), with the highest score (153) and lowest free energy (25.0 kcal mol^-1^), may be a potential target of miR-122 in tilapia. Therefore, in this study, our aims were to explore the regulatory relationship between miR-122 and *SCD*, and to determine how miR-122 regulates *SCD* expression and participates in fat regulation.

Stearoyl-CoA desaturase is a key enzyme that catalyzes the formation of n-9 monounsaturated fatty acids (MUFAs) in the ninth carbon chain of saturated fatty acids (SFAs). It is a transmembrane protein that is located in the endoplasmic reticulum, and it plays an important role in lipid metabolism in mammals (Ntambi and Miyazaki, 2004). Mice fed high-carbohydrate diets showed a significant increase in hepatic *SCD* expression, and a return to a normal diet led to a rapid decrease in *SCD* expression levels within 24 h (Ntambi, 1992). Mice fed polyunsaturated fatty acid (PUFA)- and cholesterol-rich diets also showed increased hepatic *SCD* expression (Kim et al., 2002). Compared with wild-type, SCD-1^(-/-)^ knockout mice showed reduced fat accumulation, increased insulin sensitivity, and resistance to weight gain when fed a high-fat diet (Ntambi et al., 2004). In Chinese mitten crabs (*Eriocheir sinensis*) fed on fish oil, soybean oil, or a 1:1 fish oil: soybean mixture, the *SCD* transcript levels were 3.5-, 2-, and 1.3-times higher, respectively, than that in the control group (fed a diet containing 3% base fat) (Guo et al., 2013). The addition of coconut oil (with a high proportion of SFA and a lower proportion of PUFA) to the diet significantly increased *SCD* transcript levels and increased lipid accumulation in hybrid tilapia (*O. aureus* ♂ × *O. niloticus* ♀) (Hsieh et al., 2007) and GIFT (Qiang et al., 2017b). Together, these results suggested that *SCD* also plays an important role in the regulation of fat metabolism in aquatic animals.

The aims of this study were as follows: (1) to verify that *SCD* is a potential target of miR-122; (2) to explore the regulatory relationship between miR-122 and *SCD*; and (3) to analyze the effects of inhibiting miR-122 expression on hepatic lipid metabolism and fat deposition in GIFT fed a high-fat diet. The overall aim of our research is to understand the molecular mechanisms of fat deposition and the treatment of nutritional diseases in GIFT, and to enhance protection of the liver against stress.

## Materials and Methods

The study protocols were approved by the Freshwater Fisheries Research Center of the Chinese Academy of Fishery Sciences (Wuxi, China). The GIFT juveniles were maintained in well-aerated water and anesthetized by injecting 0.01% tricaine methanesulfonate (Sigma, St. Louis, MO, United States). Liver tissue and blood were collected based on the Guide for the Care and Use of Laboratory Animals in China.

### Experimental Fish

The experimental fish were selected from the “Zhongwei No. 1” GIFT juveniles at the Yixing Base of the Freshwater Fisheries Research Center, Chinese Academy of Fishery Sciences. Fish with no disease, no injury, and strong vigor were selected. Before the experiment, the fish were kept in an indoor cement pool (water temperature 28 ± 1°C, pH 7.4 ± 0.2) for 10 days under a natural photoperiod and with continuous aeration. Submerged feed (crude protein 29.0% and crude lipid 8.0%) was provided at 8:00 and 15:00 every day, at a rate of 6% of body weight.

### Identification of miR-122 Potential Target Gene: *SCD*

First, we synthesized the 3′-untranslated region (UTR) based on the sequence of the tilapia *SCD* in GenBank (XM_003441797). The 3′-UTR fragment of the synthesized *SCD* and the pGL3-promoter vector were digested using *XbaI*, and the purified *SCD*-3′-UTR fragment and the vector pGL3-promoter were then ligated with T4 ligase. The recombinant vector was transferred into competent cells (DH5α) in a plate culture, positive monoclonal amplification was performed, and then a subsample was sent to Suzhou Jinweizhi Biotechnology Co., Ltd. (Suzhou, China) for sequencing. The sequencing results were analyzed by BLAST and screened for positive vectors containing the target genes. The constructed *SCD* 3′-UTR-luciferase reporter vector was transfected into the HEK293T cell line to analyze the potential binding sites of the miRNA to its target gene. To construct pGL3-*SCD* mutants, the six complementary bases that pair with miR-122 in the 3′-UTR region of *SCD* were deleted, six new bases were added, and the luciferase reporter gene was inserted downstream as the reporter. In the 24 h before transfection, HEK293T cells were cultured in 12-well cell plates (1.0 × 10^6^ cells/well). Then, 25 ng of the luciferase reporter vector containing 50 nM miR-122 mimic, miR-122 wild-type (wt), or the 3′-UTR mutant was co-transfected with 5 ng *Renilla luciferase* control vector (pRL-TK, Promega, Madison, WI, United States) using lipofectamine 2000 (Invitrogen, Carlsbad, CA, United States). The activity of transfected cells in each well was normalized based on the *Renilla* luciferase activity. At 36 h after transfection, the cells were washed with pre-chilled phosphate-buffered saline (PBS) and then collected by centrifugation at 800 *g* for 5 min at 4°C. A liquid scintillation counter was used to detect luciferase activity. The ability of the miRNA to bind to its target gene was indicated by the strength of luciferase activity.

### Analysis of Regulatory Relationship Between miR-122 and *SCD*

Healthy GIFT (each about 10 g) were selected and placed in 600-L tanks, each containing 20 fish. Each treatment had three replicates. The chemically synthesized miRNA antagomir was dissolved in PBS, and administered intravenously to GIFT at a dose of 50 mg kg^-1^ body weight. At the same time, the negative control (NC) or PBS (control) was injected into the tail vein of other GIFT at the same dose. At 0, 24, 48, and 72 h after treatment, liver tissues were collected from three GIFT in each tank (9 samples/treatment), immediately frozen in liquid nitrogen, and stored at -80°C until analysis.

The coding sequence and 3′-UTR of *SCD* were synthesized according to sequence information in GenBank. The fragments and pEGFP-C1 vector were digested and linearized with *HindIII* and *BamHI* endonucleases. The linearized fragment and vector were ligated using T4 ligase and then transformed into DH5α competent cells. The transformed cells were incubated at 37°C for 1 h, then uniformly spread on Luria-Bertani (LB) solid plates. The cultures were kept in a 37°C incubator overnight to screen for kanamycin resistance. Colonies that grew well on the plates were selected, and positive clones were identified by colony PCR using specific primers. The positive colonies were added to LB liquid medium containing kanamycin and cultured at 37°C overnight, and then the target plasmid was extracted with a plasmid extraction kit.

Liver tissue was dissected from healthy GIFT under aseptic conditions. The liver cells were isolated and purified according to the method of Pierce et al. (2011), and then incubated at 27°C under 5% CO_2_. When the primary cultured hepatocytes had grown by 80–90%, 2 μg of the target plasmid pEGFP-C1-3Flag-SCD-3′-UTR and 50 or 100 nm miR-122 mimic or the same dose of the miR-122 NC were, respectively, constructed and co-transfected into hepatocytes using lipofectamine 2000. The cell culture plates were observed under a fluorescent inverted microscope (Olympus X71, Olympus, Tokyo, Japan) at 48 and 72 h after transfection. Hepatocytes were collected for analysis of SCD protein levels. The exogenous expression of SCD was confirmed by western blotting using an anti-Flag monoclonal antibody.

### Role of *SCD* Targeted by miR-122 in Lipid Metabolism

Juvenile GIFT with average size 3.8 ± 0.4 g were randomly assigned to nine 600-L tanks, each containing 30 fish (270 fish in total). Each treatment had three replicates. The synthesized miRNA antagomir fragment and the miRNA NC were dissolved in PBS and injected via the tail vein at a dose of 50 mg kg^-1^ body weight on days 1, 7, 13, 19, 25, 31, and 37 (every 6 days during the 40-day experiment). In addition, three groups of experimental fish were selected and the same dose of PBS was injected into the tail vein at the same times as a control. At 48 h after the first injection, each experimental group began to be fed a high-fat diet (17% fat), formulated according to Tao et al. (2017). The liver tissues of three fish were collected randomly from each tank at 0, 10, 20, and 40 days after injection, immediately frozen in liquid nitrogen, and stored at -80°C until analysis. At the end of the experiment, feeding was stopped 24 h prior to weighing all remaining fish (15–18 fish) in each tank. The complete livers of two fish per tank were collected for microscopic analyses. Sections were stained with 0.5% oil red O stain and hematoxylin-eosin to identify the levels of fat deposition in the liver. In addition, three fish were randomly selected from each tank (nine samples per treatment) for blood biochemical analyses. After deep anesthesia, blood samples were taken from the tail vein, and then kept at 4°C for 2 h. Serum was prepared by centrifugation at 3500 × *g* for 10 min at 4°C, and the supernatant was stored at -80°C until analysis. Six fish were selected from each group to determine fatty acid composition in the liver. The livers were dissected out, lyophilized at -60°C, and then the fatty acid composition was determined as described by He et al. (2015).

### Hematoxylin and Eosin Staining and Oil Red O Staining

The liver tissues were fixed in 4% paraformaldehyde for 4 days, washed several times with PBS, and dehydrated using an alcohol gradient. Xylene was used to make the tissues transparent before embedding in paraffin. The paraffin-embedded samples were cut into 5-μm slices, dewaxed, and then stained with hematoxylin for 7 min. The slices were washed with tap water and then with warm water for 1 min, immersed in 1% hydrochloric acid for 60 s, and then stained with eosin for 5 min. The slices were dehydrated using an alcohol gradient, treated with xylene to make them transparent, and then sealed with neutral resin. Liver pathological changes were observed under a CX31 microscope (OLYMPUS, Japan).

#### Oil Red O Staining

The liver was fixed in 4% paraformaldehyde for 4 days, washed twice in PBS, and then soaked in 30% sucrose solution (diluted with PBS) overnight at 4°C. The liver was then embedded in OCT compound (Ames Division; Miles Laboratories, Elkhart, IN, United States) and sliced into 8-μm thick slices using a freezing microtome. The slices were heated at 60°C for 30 min, infiltrated with 85% 1,2-propylene glycol for 5 min and then with 100% 1,2-propylene glycol for 5 min, and then stained with freshly prepared 0.5% oil red O at room temperature for 2 h. The staining solution was rinsed off with PBS solution, and the sample was immersed in 100% 1,2-propanediol for 1 min to remove the background color. The propylene glycol was washed off with PBS, and then the sample was counterstained with hematoxylin for 10 s. After washing with water, the slides were dried and sealed with gelatin. The red-colored lipid droplets were observed and photographed under a microscope.

### Analyses of Gene and Protein Expression and Biochemical Parameters

#### miR-122 Expression

Specific primers for miR-122 are shown in **Table 1**. Extraction of miRNAs, synthesis of first-strand cDNA, and qRT-PCR analyses were conducted using the procedures described by Qiang et al. (2017b). We amplified U6 as the reference gene, using the U6 primer supplied in the miRNA SYBR Green qRT-PCR kit (Takara, Dalian, China).

**Table 1:** Primer sequences.

miRNA or mRNA	Sequence
miR-122	CTGGAGTGTGACAATGGTGTTT
SCD	F: 5′-ACAAGCTCTCCGTGCTGGTCAT-3′
	R: 5′-GCAGAGTTGGGACGAAGTAGGC-3′
LPL	F: 5′-TACACGGCTGGACGGTAACAG-3′
	R: 5′-AGGTCGGGTAGTGCTGATTGG-3′
CPT1	F: 5′-AGAGGCCGTGGACCTATCAT-3′
	F: 5′-GAGGTGGGGAACACGTACAG-3′
SREBP-1	F: 5′-GCGCATTTCATGAGGCGAAT-3′
	F: 5′-TAACCCCGGTCTATGGAGCA-3′
PPARα	F: 5′-TCCAAAAGAAGAACCGAAACA-3′
	F: 5′-TTCCACCTCTTTCTCAACCAT-3′
18S rRNA	F: 5′-GGCCGTTCTTAGTTGGTGGA-3′
	F: 5′-TTGCTCAATCTCGTGTGGCT-3′

#### Analysis of Lipid Metabolism-Related Gene Expression

The specific primers used to amplify *SCD* are shown in **Table 1**. Extraction of total RNA, the RT reaction, and qRT-PCR analyses were conducted as described by Qiang et al.(2017b). To test the potential role of miR-122 in regulating lipid metabolism in fish fed a high-fat diet, the transcript levels of genes encoding carnitine palmitoyl transferase 1 (*CPT1*), lipoprotein lipase (*LPL*), peroxisome proliferator-activated receptor alpha (*PPARα*), and sterol regulatory element-binding protein 1 (*SREBP-1*) were analyzed by qRT-PCR using the same procedure as that used to amplify *SCD*. The primers used are shown in **Table 1**, and 18s rRNA was the reference gene.

#### Analysis of SCD Protein Levels by Western Blotting

The cell culture medium was discarded, and the cells were washed twice with PBS. An appropriate amount of 1× lysis buffer was added, and then the cells were transferred to an Eppendorf tube and lysed at 4°C for 30 min. After centrifugation at 12000 × *g* for 15 min at 4°C, the protein supernatant was aspirated, and 5× sodium dodecyl sulfate (SDS) protein loading buffer was added. The proteins were denatured by heating at 100°C for 10 min, separated by sodium dodecyl sulfate polyacrylamide gel electrophoresis (SDS-PAGE), and then transferred onto a polyvinylidene fluoride membrane using the wet transfer method. The membranes were blocked in 5% (w/v) skim milk powder for 3 h, washed with Tris buffered saline with Tween (TBST), and then incubated with the primary antibody: Flag (Shanghai, China) at 4°C overnight. On the second day, the membranes were washed with TBST and incubated with the corresponding secondary antibody: rabbit IgG (Cell Signaling Technology, Danvers, MA, United States) for 1 h at room temperature. The color was developed using Immobilon Western HRP luminescence reagent (Millipore, Billerica, MA, United States). The reference protein was GAPDH.

#### Analysis of Serum or Hepatic Triglyceride (TG) and Cholesterol (TC) Contents

Serum TG and TC were analyzed as described by Richmond (1973) and Schettler and Nussel (1975), respectively, and were measured using a Roche Cobas C311 automatic biochemical analyzer (Roche, Basel, Switzerland). Hepatic lipids were extracted according to the classical method of Folch et al. (1957). Hepatic TG and TC were analyzed as described by Yun et al. (2011) and Wang et al. (2017), respectively, and were quantified by enzyme-linked immunosorbent assay (ELISA) using test kits. All assay kits used were obtained from Shanghai Lengton Bioscience (Shanghai, China).

### Statistical Analysis

The relative expression levels of mRNA and miRNA were calculated assuming that the qPCR amplification efficiency of gene expression and reference genes were basically the same. The relative expression levels of mRNA and miRNA in each experimental group were determined using the 2^-ΔΔCT^ method based on the relative expression level in the experimental control group at 0 h. The data are expressed as mean ± standard deviation (SD). Experimental data were subjected to variance analysis using SPSS 17.0 statistical software. Data for the same experimental group at different sampling times were compared by paired-sample *t* test, and significant differences are indicated by asterisks above histograms. Different treatment groups at each sampling time were compared using Duncan’s multiple comparisons, and significant differences are indicated by different lowercase letters above histograms. Differences were considered significant at *P* < 0.05.

## Results

### Analysis of miR-122 Potential Target Gene in GIFT Liver

**FIGURE 1:**
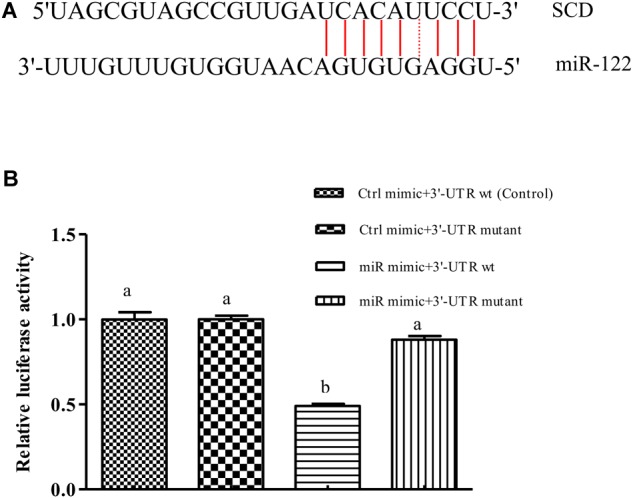
miR-122 regulates SCD expression by binding with 6-nucleotide sequence in *SCD* 3′-UTR. **(A)** Sequence complementarity between miR-122 and *SCD* 3’-UTR. **(B)** Dual luciferase reporter assay system used to test binding between miR-122 and *SCD* in HEK293T cells. Ctrl mimic, 3′-UTR wt, 3′-UTR mutant, and miR mimic are also referred to as miRNA negative control, SCD-WT, SCD-Mut, and miR-122 mimic, respectively. Different lowercase letters show significant differences among treatments at each sampling point (*P* < 0.05, Duncan’s multiple range test).

We used bioinformatics software to predict the binding site of miR-122 in the GIFT *SCD* 3′-UTR. The seed region (bases 2–8) of miR-122 and the sequence at 1891–1916 bp in the 3′-UTR of *SCD* were complementary (**Figure 1A**), and the binding free energy was very low (-25.0 kcal mol^-1^). This suggested that miR-122 has a potential binding site in *SCD*.

The results of the double luciferase reporter assay showed that the miR-122 mimic significantly reduced the luciferase activity of the pGL3-*SCD-*3′-UTR vector (*P* < 0.05) (**Figure 1B**), while it did not affect the luciferase activity of the mutant *SCD* 3′-UTR (*P* > 0.05). In addition, there was no significant difference in luciferase activity among miR-122 NC+*SCD*-3′-UTR wt, the miR-122 NC+*SCD*-3′-UTR mutant, and the miR-122 mimic+*SCD*-3′-UTR mutant (*P* > 0.05), suggesting that miR-122 binds to a site in the *SCD*-3′-UTR sequence.

### Analysis of Regulatory Relationship Between miR-122 and *SCD*

**FIGURE 2:**
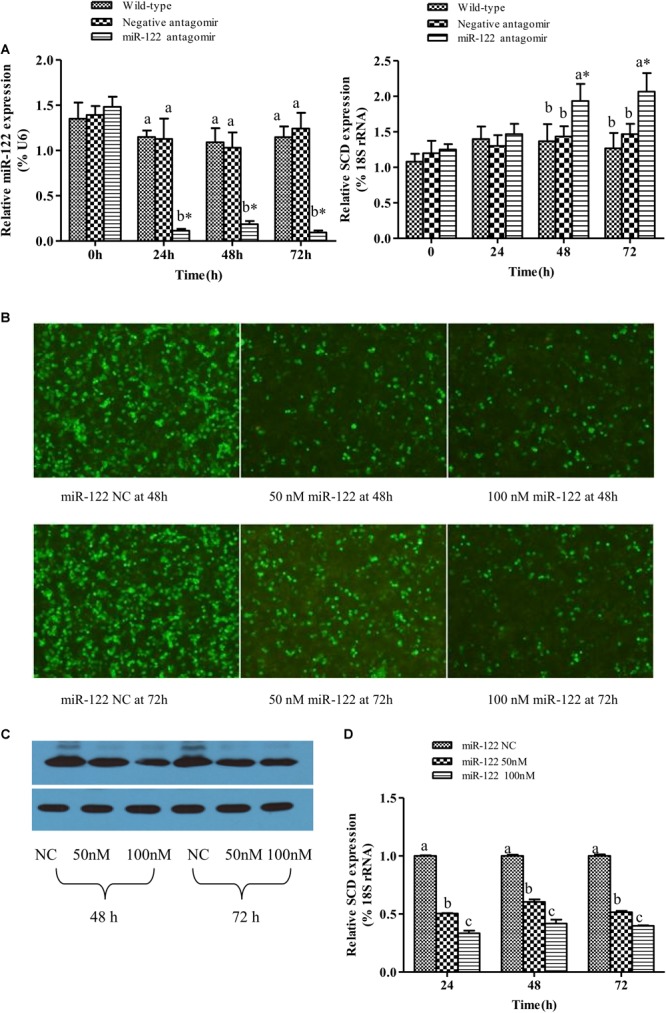
Analysis of regulatory relationships between miR-122 and *SCD*. **(A)** Juvenile GIFT weighing about 10 g were injected in tail vein with PBS, miR-122 negative control, or miR-122 antagomir (dose, 50 mg kg^-1^ body weight). Transcript levels of *SCD* and miR-122 in GIFT liver were determined by qRT-PCR for 72 h. GIFT injected with PBS served as control. **(B)** Verification of regulatory relationship between miR-122 and *SCD* at hepatocyte level. pEGFP-C1-3Flag-SCD-3′-UTR and 50 nM or 100 nM miR-122 mimic or same dose of miR-122 negative control was co-transfected into hepatocytes by lipofectamine 2000. **(C)** Protein expressions of SCD at hepatocyte level as determined by western blotting. **(D)** Transcript levels of *SCD* at hepatocyte level as determined by qRT-PCR. ^∗^*P* < 0.05 indicates significant differences between values obtained before and after injection or transfection (paired-samples *t* test). Different lowercase letters show significant differences among treatments at each sampling point (*P* < 0.05, Duncan’s multiple range test).

As shown in **Figures 2A**, the GIFT injected with miR-122 antagomir showed significantly inhibited miRNA expression at 24, 48, and 72 h. However, there was no significant difference in miR-122 expression between the control and NC groups at 24, 48, and 72 h (*P* > 0.05). These groups showed significantly higher miR-122 expression than that of the miR-122 antagomir group (*P* < 0.05).

At 0 and 24 h, there was no significant difference in *SCD* transcript levels among the treatment groups (*P* > 0.05). At 48 and 72 h, *SCD* transcript levels were significantly higher in the miR-122 antagomir group than in the control and NC groups (*P* < 0.05) (**Figures 2A**).

When the target plasmid was transfected into hepatocytes, the luciferase activity was significantly lower in the groups co-transfected with 50 or 100 nM miR-122 mimic than in the control group at 48 and 72 h (*P* < 0.05) (**Figure 2B**). The protein expression levels of SCD in the miR-122 mimic groups (50 and 100 nM) were also reduced at 48 and 72 h (*P* < 0.05) (**Figure 2C**). The qRT-PCR analysis showed that the transcript levels of *SCD* were significantly lower in the 50 and 100 nm miR-122 mimic groups than in the control group (**Figure 2D**). These results indicated that up-regulation or down-regulation of miR-122 expression could reverse the expression of *SCD* (*P* < 0.05).

### Analysis of Regulatory Function of miR-122 Targeting *SCD* in Lipid Metabolism

**FIGURE 3:**
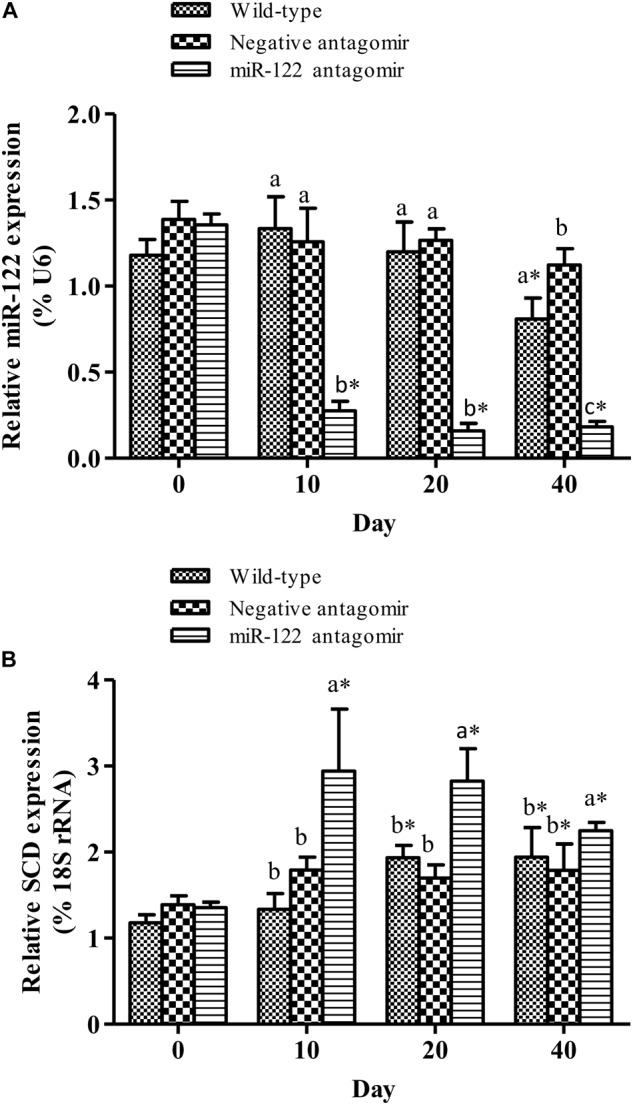
Effect of inhibition of miR-122 on hepatic *SCD* expression in GIFT. Juvenile GIFT weighing about 3.8 g were injected in tail vein with PBS, miR-122 negative control, or miR-122 antagomir (dose, 50 mg kg^-1^ body weight) every 6 days for 40 days. GIFT injected with PBS served as control. **(A)** The expression of miR-122 in liver was detected on days 0, 10, 20, and 40 using qRT-PCR, with U6 as the reference gene. **(B)** The expression of SCD in liver was detected on days 0, 10, 20, and 40 using qRT-PCR, with 18S rRNA as the reference gene. ^∗^*P* < 0.05 indicates significant differences in each group among sampling points (paired-samples *t* test). The data were expressed as the relative change compared with the control group on day 0. Different lowercase letters show significant differences among treatments at each sampling point (*P* < 0.05, Duncan’s multiple range test).

**FIGURE 4:**
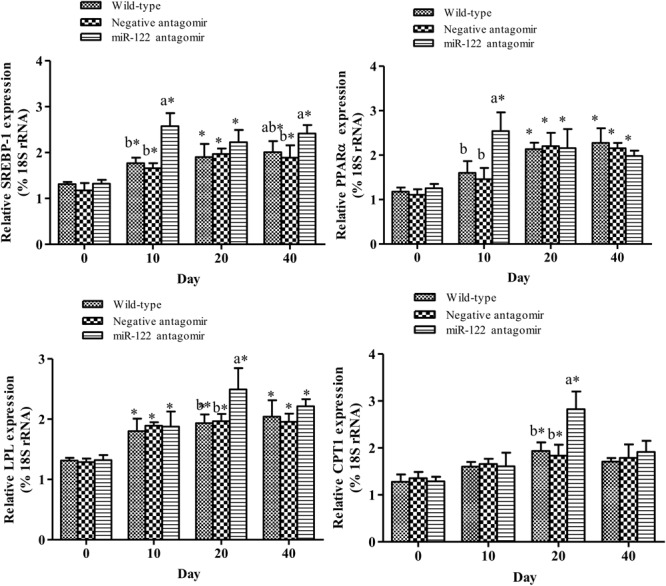
Effect of hepatic *SCD* up-regulation on expression of genes related to lipid metabolism in GIFT. GIFT injected with PBS served as control. ^∗^*P* < 0.05 indicates significant differences in each group among sampling points (paired-samples *t* test). The data were expressed as the relative change compared with the control group on day 0. Different lowercase letters show significant differences among treatments at each sampling point (*P* < 0.05, Duncan’s multiple range test).

To further analyze the effect of miR-122 expression on lipid regulation in GIFT, we investigated the effect of down-regulation of miR-122 on hepatic lipid metabolism-related genes and fat deposition under high-fat diet stress. The injection of miR-122 antagomir significantly inhibited miR-122 expression in GIFT liver during the 40-day experiment (**Figure 3A**). The expression level of miR-122 in the control group was significantly lower at 40 days than at 0, 10, and 20 days under high-fat diet stress (*P* < 0.05). At 10 and 20 days, the *SCD* transcript levels were significantly higher in the miR-122 antagomir group than in the control and NC groups (*P* < 0.05). The *SCD* transcript levels in the control and NC groups also showed a gradually increasing trend for 40 days. However, the *SCD* transcript level in the miR-122 antagomir group was significantly lower at 40 days than at 10 and 20 days (**Figure 3B**). In terms of other lipid-metabolizing enzymes, the transcript levels of *CPT1, LPL, SREBP-1*, and *PPARα* in the liver of each experimental group tended to increase under high-fat diet stress (**Figure 4**). The transcript levels of *CPT1* and *LPL* were significantly higher in the miR-122 antagomir group than in the other groups at 20 days (*P* < 0.05), and those of *SREBP-1* and *PPARα* were significantly higher in the miR-122 antagomir group than in the other groups at 10 days (*P* < 0.05).

**FIGURE 5:**
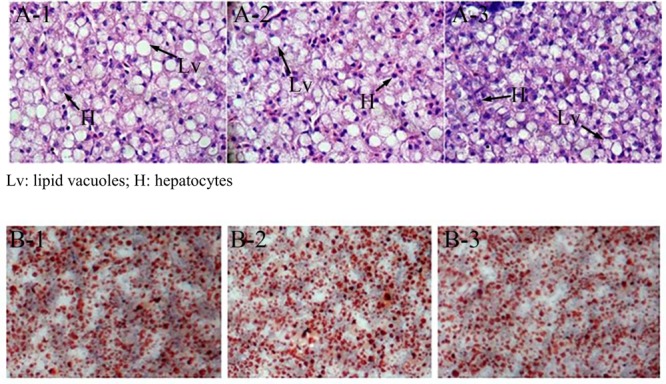
Effect of miR-122 inhibition on fat deposition in liver tissue of GIFT fed a high-fat diet as determined by hematoxylin and eosin staining and oil red O staining. **(A-1,B-1)** Wild-type groups; **(A-2,B-2)** negative control groups; **(A-3,B-3)** miR-122 antagomir groups.

At the end of the 40-day experiment, the final body weight and TG content of tilapia were significantly higher in the miR-122 antagomir group than in the control and NC groups; the TC was also significantly lower in the antagomir group than in the control group (*P* < 0.05) (**Table 2**). There were no significant differences in serum TC and TG levels among the experimental groups (*P* > 0.05). The hematoxylin and eosin staining analysis revealed many lipid vacuoles in the liver tissue of each experimental group (**Figures 5A-1–A-3**). Many red lipid droplets were also observed by oil red O staining (**Figures 5B-1–B-3**). The fatty acid composition analysis showed that the higher ratios of MUFA/SFA and the lower ratios of SFA/unsaturated fatty acid (UFA) in the liver were found in the miR-122 antagomir group compared with the control and NC groups.

**Table 2:** Growth, biochemical parameters, and fatty acid profiles of juvenile GIFT injected with PBS, negative antagomir, or miR-122 antagomir every 6 days for 40 days.

	PBS	Negative antagomir	miR-122 antagomir
**Growth parameters (*n* = 30 replicates per group)**
Initial fish weight (g)	3.22 0.24	3.41 0.16	3.29 0.11
Final fish weight (g)	29.25 2.43^b^	31.26 1.58^a^b	35.16 2.18^a^
WG (%)	8.08 0.71^b^	8.17 0.63^b^	9.67 0.74^a^
**Liver parameters(*n* = 9 replicates per group)**
TG (mmol⋅L^-1^)	13.56 1.32^b^	14.17 1.27^b^	16.78 1.72^a^
TC (mmol⋅L^-1^)	5.13 0.73^b^	5.66 0.52^a^b	6.12 0.41^a^
**Serum parameters(*n* = 9 replicates per group)**
TG (mmol⋅L^-1^)	3.68 0.25	4.03 0.37	3.73 0.26
TC (mmol⋅L^-1^)	4.15 0.24	3.73 0.21	3.84 0.19
**Fatty acid profiles(*n* = 6 replicates per group)**
MUFA/SFA	0.63 0.02^a^	0.68 0.02^a^	0.82 0.02^b^
SFA/UFA	1.12 0.11^a^	1.08 0.09^a^	0.96 0.18^b^

*WG, weight gain; TC, cholesterol; TG, triglyceride; SFA, saturated fatty acid; MUFA, monounsaturated fatty acid; UFA, unsaturated fatty acid. Different lowercase letters indicate significant differences (P < 0.05) within different groups (Duncan’s multiple range test).*

## Discussion

miRNAs are a class of non-coding small RNAs approximately 22 nt in length, which bind to complementary sequences in their target genes to negatively regulate their post-transcriptional levels. In mammals, miRNAs are involved in lipid metabolism at many levels, including adipocyte differentiation, adipose tissue energy metabolism, hepatic lipid metabolism, and secretion of hormones related to the regulation of lipid metabolism (Giral et al., 2016). Many miRNAs including miR-30c, miR-122, miR-148a, miR-27a/b, miR-185, and miR-92a are known to be involved in the regulation of lipid metabolism. Silencing of miR-122 in hepatocytes was shown to down-regulate the expression of SOCS3 (suppressor of cytokine signaling), which impaired the inhibition of the STAT3 (signal transducer and activator of transcription activity 3), leading to reduced cholesterol levels (Shibata et al., 2013). However, miR-122 expression could be restored by forced expression of SOCS3, thereby increasing cholesterol and fatty acid levels. Tsai et al. (2012) produced a mutant mouse strain (miR-122^-/-^mice) with a germline-deleted miR-122a by a homologous recombination method. Compared with wild-type mice, the mutant mice showed significantly lower serum low-density cholesterol levels. Further studies showed that the loss of miR-122 led to decreased levels of microsomal triglyceride transfer protein, resulting in blocked synthesis of low density cholesterol (Tsai et al., 2012).

Down-regulation of miR-122 expression under high-fat diet stress may play an important role in lipid metabolism in GIFT liver. *In vivo*, miRNAs usually interact with the 3′-UTR region of the target gene’s mRNA. This regulation of gene expression exerts biological effects. We used bioinformatics analyses and a dual luciferase reporter assay system to verify the binding site between miRNA-122 and its potential target gene at the cellular level. We identified a sequence at positions 2–8 in the *SCD* 3′-UTR that was fully complementary to the 5′-end of the miR-122 seed region. With the overexpression of miR-122, the relative activity of the luciferase reporter linked to the target *SCD* in HEK293T was significantly decreased. We mutated the 6-base site of the 3′-UTR of *SCD* that was complementary to the miR-122 seed region and constructed a mutation reporter vector of the target site. miR-122 could not inhibit the activity of the luciferase reporter linked to *SCD* after mutation of the 6-base site, confirming that it is the binding site between *SCD* and miR-122.

In the same miRNA family, even if different precursors produce the same or similar miRNA mature sequences, these sequences may target different genes and have different regulatory effects, resulting in different temporal and spatial gene expression patterns, because of differences in their upstream regulatory sequences. In human hepatic stellate cells, during the self-renewal of embryonic stem cells, and during hepatocellular carcinoma proliferation, miR-122 can regulate the expression of target genes (encoding the prolyl 4-hydroxylase subunit alpha-1 precursor and pyruvate kinase isozymes) to participate in the corresponding biological processes (Jung et al., 2011; Li et al., 2013). miR-122 has been relatively highly conserved in different species during evolution. The miR-122 sequence in GIFT is completely identical to that in mammals, and is also highly enriched in the liver, suggesting that miR-122 also has important biological activity in fish (Qiang et al., 2017c). In this study, the miR-122-mediated target gene *SCD* involved in lipid metabolism was analyzed to reveal how miR-122 regulates lipid metabolism in GIFT liver.

*SCD* encodes a key enzyme that catalyzes the formation of MUFAs from SFAs. In the process of fat synthesis, MUFAs are more likely than SFAs to become substrates of acyl-CoA cholesterol acyl transferase and diacylglycerol acyltransferase, which produce cholesterol and triglycerides, respectively (Ntambi, 1999). Ntambi et al. (2010) found that the expression of *GULT-1* (encoding glucose transporter 1) was up-regulated in adipose tissue with knocked-out *SCD-1*, suggesting that *SCD-1* is related to glucose metabolism in adipose tissue. The thermogenic effect of skeletal muscle is related to SCD-1 activity. Overexpression of SCD-1 was shown to reduce the thermogenic effect of skeletal muscle, and the mechanism of decreased heat production may have been related to the reduction in fatty acid oxidation (Mainieri et al., 2006). The liver is the most metabolically active organ in animals and plays an important role in maintaining the dynamic balance of energy metabolism. The liver is also the main site for fat synthesis in fish, accounting for more than 90% of the total fat synthesized. Our results showed that inhibition of miR-122 promoted the expression of *SCD* in GIFT liver. Highly expressed *SCD* may regulate fish growth and lipid metabolism.

Under laboratory conditions, largemouth bass (*Micropterus salmoides*) fed a high-fat diet showed increased TC and low-density lipoprotein levels and a reduced ability to transport and utilize fat (Zhu et al., 2018). Barracuda (*Chelon haematocheilus*) fed a high-fat diet (16.9%) showed increased lipolytic enzymes activity and serum TG and TC levels (Zhang et al., 2013). GIFT larvae fed a high-fat diet (18.5%) also showed growth inhibition, increased body fat deposition, and increased susceptibility to streptococcal infection (Qiang et al., 2017a). In this study, the growth and hepatic TC and TG were significantly higher in the miR-122 antagomir group than in the control group when the fish were fed a high-fat diet. Higher *SCD* expression levels in the liver may have altered the content of MUFAs in cells and promoted the synthesis of TG and TC precursors. However, the levels of serum TC and TG in the antagomir group did not increase with up-regulated *SCD* expression. Blood transports nutrients and metabolic wastes in animals, and blood lipid levels can reflect fat metabolism. If the structure or function of liver cells is impaired, serum TC and TG levels could rise dramatically. Lin et al. (1990) also found that the lipid content of grass carp (*Ctenopharyngodon idella*) with fatty liver disease was negatively related to the serum TG content. In this study, we observed extensive fat deposition in each experimental group by oil red O staining and hematoxylin and eosin staining. However, the liver tissue appeared to be undamaged, and the fat metabolism function may have been only slightly or not impaired. This may explain why there were no changes in serum TC and TG levels.

Up-regulation of hepatic *SCD* expression can promote the expression of some lipid metabolism-related genes. In mammals, stimulating transcription factors such as *SREBP-1* and *PPARα* led to increased fat content in the liver (Dossi et al., 2014). *SREBP-1* is a central transcription factor involved in lipid metabolism. Increased SREBP activity was shown to activate the expression of more than 30 genes related to the synthesis and uptake of cholesterol, fatty acids, triglycerides, and phospholipids, and increase the synthesis of molecules required for NADPH cofactors. This led to TC and fatty acid accumulation, and consequently, increased lipid levels (Shibata et al., 2013). *PPARα* is highly expressed in fatty acid metabolism-rich tissues such as brown fat, liver, heart, and skeletal muscle (Braissant et al., 1996). In this study, the down-regulation of miR-122 expression promoted the expression of hepatic *SCD, SREBP-1*, and *PPARα* at 10 days, thereby enhancing lipid metabolism. However, at 40 days of high fat-diet stress, the expression of *SCD* was significantly down-regulated in the miR-122 antagomir group. The same target gene may be regulated by many miRNAs, and there may be compensatory effects among different miRNAs. Therefore, to maintain homeostasis *in vivo*, miRNAs may have different temporal and spatial effects on the regulation of target genes. Inhibition of miRNA expression could promote the expression of target genes to a certain extent, but it is also possible to activate other miRNAs that target the same gene. The specific regulatory mechanisms remain to be further studied. In this study, the *SCD, SREBP-1*, and *PPARα* transcript levels in the control group increased during the 40-days experiment, which may be attributed to changes in fat metabolism and adaptation mechanisms in GIFT liver.

One of the key enzymes in the metabolism of lipoproteins is LPL. Studies in both fish and mammals have shown that LPL is closely related to lipid metabolism in the body and obesity. In red seabream (*Pagrus major*) (Liang et al., 2002) and yellow catfish (*Pelteobagrus fulvidraco*) (Qiang et al., 2017d), LPL plays a major role in regulating lipid metabolism. When these fish were fed a high-fat diet, more lipid substrate was sent to the liver and consumed by accelerated oxidation. Under high-fat diet stress, the increased hepatic TG and TC of GIFT may have enhanced lipid metabolism, and thus promoted the expression of *LPL*. At 20 days of stress, the higher transcript level of *CPT1* (encoding a lipolytic enzyme) in the miR-122 antagomir group may have led to increased oxidative breakdown of excess fat, resulting in reduced fat deposition. With prolonged exposure to a high-fat diet, GIFT may become adapted to this type of diet and reduce expression of *CPT1.* This would increase hepatic fat accumulation, thereby increasing hepatic TG and TC levels (Matsumoto et al., 2010).

## Conclusion

In this study, we verified that there is a binding site between miR-122 and a sequence in the 3′-UTR of *SCD* in GIFT. Inhibition of miR-122 up-regulated *SCD* and interfered with the regulation of lipid metabolism. Under high-fat diet stress, up-regulation of *SCD* helped to promote the expression of genes related to fat synthesis (*SREBP-1* and *LPL*) and increased the TC and TG contents in the liver. The results of this study provide new insights into the regulatory mechanisms of fat deposition in GIFT liver.

## Author Contributions

PX and JQ conceived and designed the experiment. JB and YT verified the binding site. JH carried out the functional analysis of miRNA. JB isolated and cultured the cells. HL conceived and implemented the database. DC analyzed the serum and hepatic indicators. JQ wrote the paper with contributions from YT, JB, PX, JH, and DC. All authors read and approved the final version of the manuscript.

## Conflict of Interest Statement

The authors declare that the research was conducted in the absence of any commercial or financial relationships that could be construed as a potential conflict of interest.

## Footnotes

1 http://www.ncbi.nlm.nih.gov/genome/?term=Oreochromis%20niloticus

2 http://www.targetscan.org/vert_50/

3 http://www.microrna.org/microrna/home.do
